# Tobacco Poisoning

**Published:** 1885-02

**Authors:** L. G. Hardman

**Affiliations:** Harmony Grove, Ga.


### TOBACCO POISONING.

By L. G. HARDMAN, M. D., Harmony Grove, Ga.

---

Mrs. W., age about 40, a woman of medium size, fair complexion, moderate health, a seamstress, has suffered at times with neuralgia. Has been a constant user of tobacco for thirty years, chewing, smoking and snuffing, all of which she continued until a short time ago, when she was advised to leave off smoking as it was believed to be the cause of her neuralgia. This she did, but continued to chew and use snuff. On the 5th of August, 1884, I was called to see her about one o'clock in the day. When I arrived I received this history of her case: While at the shop, at her usual vocation, with her husband, he purchased her a box of snuff between 10 and 11 o'clock in the day, and a short time afterward she left the shop and went home to look after her household affairs. She went home alone and was alone for an hour or more before any one came. About 12 o'clock her husband came home—his usual dinner hour—and, on entering the door, found her lying on the floor unconscious, almost helpless and sweating profusely. When he spoke to her she gave no answer. She was put on the bed. She had vomited freely over the room and on her own person. There was evidence that she had prepared dinner and had eaten some before she was stricken down. When I examined her I found she was unconscious; surface cold, almost pulseless, and occasionally she would move a hand or foot and groan. Respiration slow but not stertorous; inclined to sleep; could not speak or swallow. By this time she was seized with a convulsion; the convulsive action was not very marked in the lower extremities but very noticeable in the face and upper extremities, with froth in her mouth. As soon as she rallied I gave her a hypodermic injection of whisky and morphine. as her pulse was only forty per minute. After the injection the pulse soon went up to fifty two and remained at that for two hours, and in one hour more, by the constant use of whisky in small doses often repeated, was 72 beats per minute. Her pupils were dilated. (I will state that authors differ in regard to the pupils in tobacco poisoning. Taylor and Woodman, with others, hold that it is dilated in acute tobacco poisoning, while Pereira and Bartholow say it is contracted, but in chronic nicotinism it is dilated. Now this is an acute tobacco poisoning in a chronic case, just as we may have an acute attack of Bright's disease coming on in the chronic.) But to return to the description of the symptoms present. She would sometimes complain of her head paining her, and then again say nothing hurt her. I was then called away, but returned again at 8 o'clock in the evening and found her still delirious, pulse same, temperature 97½, could move about more, vomited once, micturition frequent and more than usual. Could not see well; said she could not tell who I was but seemed to know for a moment when told; also noticed facial twitching at this time. She gradually improved in every respect up to Saturday, when I saw her last. Rested well Saturday night, and doing well, so far as the family could tell, Sunday morning. She could not see well; voice weak; temperature never was elevated; pulse never over 80; no paralysis. After she was doing well as stated even on Sunday morning, I was surprised to hear, at 11 o'clock, she was dead, and on inquiring of her husband, he said about 8 o'clock she took some tobacco, and between 10 and 11 some more; she was quiet at this time and he stepped out, but very soon he heard her give one scream and he ran back and she died a few minutes thereafter.

I told the husband on my first visit it was tobacco poisoning, but I had never seen a case of it before and was not positive. After I visited her a time or two I stated to him that I was satisfied my diagnosis was correct. He did not, however, believe me, as he was a dear lover of the poisonous plant. In order that all parties might be satisfied, and that her husband might see that her stomach was not full of bile, as he supposed, and it was nothing but a bilious attack, as the doctors say, we sent for the coroner and had an autopsy. No external injury. An incision was made from zyphoid cartilage to the symphysis pubis and another at right angles to this at free margin of ribs; peritoneum and intestines, liver, kidneys and spleen normal in appearance except kidneys somewhat congested. Stomach was opened and it contained nothing but particles of tobacco; the mucous membrane of the stomach was covered with exchamosed spots but no other abnormal appearance. The last tobacco she took before her death she did not spit at all. I found, on examination of the snuffbox, she had used about one-fourth of an ordinary ounce snuffbox.

It is astonishing, when we notice how much tobacco is taken at one time by an ordinary chewer, that every chew does not produce death; it would undoubtedly do it if all the nicotine in the chew was taken into the stomach at one time. An ordinary chewer takes from 20 to 60 grains at a chew, and 15 grains has produced death, according to some authority, in fifteen minutes. While tobacco is a great luxury to some, I believe there are sudden deaths from this cause which, at times, we cannot account for; and I believe, if it was not for the great eliminating action of the kidneys, that death would result much oftener than it does. I believe tobacco a frequent cause of neuralgia and other nerve troubles.

